# Examination of a Canada-Wide Collaboration Platform for Order Sets: Retrospective Analysis

**DOI:** 10.2196/26123

**Published:** 2021-11-29

**Authors:** Arshia Pedram Javidan, Allan Brand, Andrew Cameron, Tommaso D'Ovidio, Martin Persaud, Kirsten Lewis, Chris O'Connor

**Affiliations:** 1 Division of Vascular Surgery Department of Surgery University of Toronto Toronto, ON Canada; 2 Institute of Health Policy, Management, and Evaluation University of Toronto Toronto, ON Canada; 3 Think Research Toronto, ON Canada; 4 Department of Emergency Medicine Faculty of Medicine University of British Columbia Vancouver, BC Canada; 5 Trillium Health Partners Mississauga Site Mississauga, ON Canada

**Keywords:** evidence-based medicine, health informatics, knowledge translation, order sets, Web 2.0

## Abstract

**Background:**

Knowledge translation and dissemination are some of the main challenges that affect evidence-based medicine. Web 2.0 platforms promote the sharing and collaborative development of content. Executable knowledge tools, such as order sets, are a knowledge translation tool whose localization is critical to its effectiveness but a challenge for organizations to develop independently.

**Objective:**

This paper describes a Web 2.0 resource, referred to as the collaborative network (TCN), for order set development designed to share executable knowledge (order sets). This paper also analyzes the scope of its use, describes its use through network analysis, and examines the provision and use of order sets in the platform by organizational size.

**Methods:**

Data were collected from Think Research’s TxConnect platform. We measured interorganization sharing across Canadian hospitals using descriptive statistics. A weighted chi-square analysis was used to evaluate institutional size to share volumes based on institution size, with post hoc Cramer V score to measure the strength of association.

**Results:**

TCN consisted of 12,495 order sets across 683 diagnoses or processes. Between January 2010 and March 2015, a total of 131 health care organizations representing 360 hospitals in Canada downloaded order sets 105,496 times. Order sets related to acute coronary syndrome, analgesia, and venous thromboembolism were most commonly shared. COVID-19 order sets were among the most actively shared, adjusting for order set lifetime. A weighted chi-square analysis showed nonrandom downloading behavior (*P*<.001), with medium-sized institutions downloading content from larger institutions acting as the most significant driver of this variance (chi-gram=124.70).

**Conclusions:**

In this paper, we have described and analyzed a Web 2.0 platform for the sharing of order set content with significant network activity. The robust use of TCN to access customized order sets reflects its value as a resource for health care organizations when they develop or update their own order sets.

## Introduction

### Background

There continues to be increased application of evidence-based medicine in clinical practice [1]. However, the volume of published evidence available makes direct application of the most appropriate evidence difficult to reliably apply at the point of care. Effective knowledge translation could address this problem by ensuring that physician practice reflects the best current evidence [2-4]. Knowledge transfer, a key component of knowledge translation, focuses on how information, knowledge, and resources are disseminated and exchanged among relevant clinicians. Continuing medical education (CME) is a mechanism that facilitates knowledge transfer and, more broadly, knowledge translation [4]. Despite its intuitive appeal, studies on didactic CME activities (eg, grand rounds) do not show significant changes in physician behavior [5]. Interactive CME activities can be more effective; however, their impact is limited because of narrow outreach, logistics, and cost [5-7].

Web 2.0 platforms (ie, web-based platforms that facilitate information sharing through user-generated content) allow for improved physician collaboration and knowledge translation [8,9]. For example, the Twitter Free Open Access Medical Education community generated >1 billion tweet impressions among nearly 50,000 users over 2 years [10-12]. However, these platforms predominantly focus on referential knowledge (ie, information a physician refers to through textbooks or articles) instead of executable knowledge (ie, information converted into tools used directly in patient care). The literature evaluating collaboration on these platforms has focused on platforms that primarily share referential knowledge, general use platforms (eg, Twitter), or platforms for specific specialties (eg, emergency medicine blogs) [10-15]. There have been limited studies on platforms that focus on sharing executable knowledge [9].

Order sets (collections of architected predefined orders) are a type of executable knowledge designed to deliver evidence-based best practices [16] that have been shown to improve patient care, safety, and efficiency [17-23]. Order sets are predetermined templates that represent a collection of orders specific to a particular hospital process (eg, admission to the intensive care unit) or a particular condition (eg, order set for acute coronary syndrome and order set for insulin administration in the context of diabetes or hyperglycemia). They can be either paper or electronic based and often represent best practices for the condition or process to which they pertain [17-23]. They offer benefits over traditional CME by making best practices directly actionable at the point of care in a structured format. Order sets must often be localized to meet local resource and workflow needs. This localization has typically been done on an isolated basis with no formal collaborative infrastructure among organizations. This siloed approach impedes effective knowledge transfer and, by association, knowledge translation.

### Objectives

The aim of this study is to first describe a Web 2.0 network (the collaborative network [TCN]) that enables the sharing of localized executable knowledge through order sets and clinical guidelines. The variation created through localization acts as a network attractor, as clinicians may be interested in understanding how others have translated evidence into practice. We also aim to examine the use of TCN through network analysis and the use and provision of order sets in TCN stratified by organizational size. To our knowledge, this is the first study of its kind to describe the networked sharing of executable content at this scale, focusing on a Canadian setting.

## Methods

### The Collaborative Network

TCN acts as a resource repository for all content developed by Think Research and the organizations participating in the network. In this study, we focused on the knowledge base for 3 related sets of data: reference clinical order set content developed by Think Research and adapted order set content from 2 sources—partner agencies (eg, governmental and clinical specialist groups) and local participating hospitals or health care institutions.

### Collaboration: Contribution

TCN functions as the main system for order set knowledge translation, exchange, and collaboration across and within organizations on the network. Upon developing new order sets or updating existing order sets, participating organizations can access the network order set library for any other organization. Knowledge from one organization about how orders are localized to practice can then be accessed by other organizations. Some organizations may choose to use these order sets as the starting point for development or simply reference them during the process of updating their existing order sets. The process of contribution and access creates a community of practice in which hospitals exchange ideas about the uptake of evidence-based best practices and how it is implemented.

### Collaboration: Access

**Figure 1:**
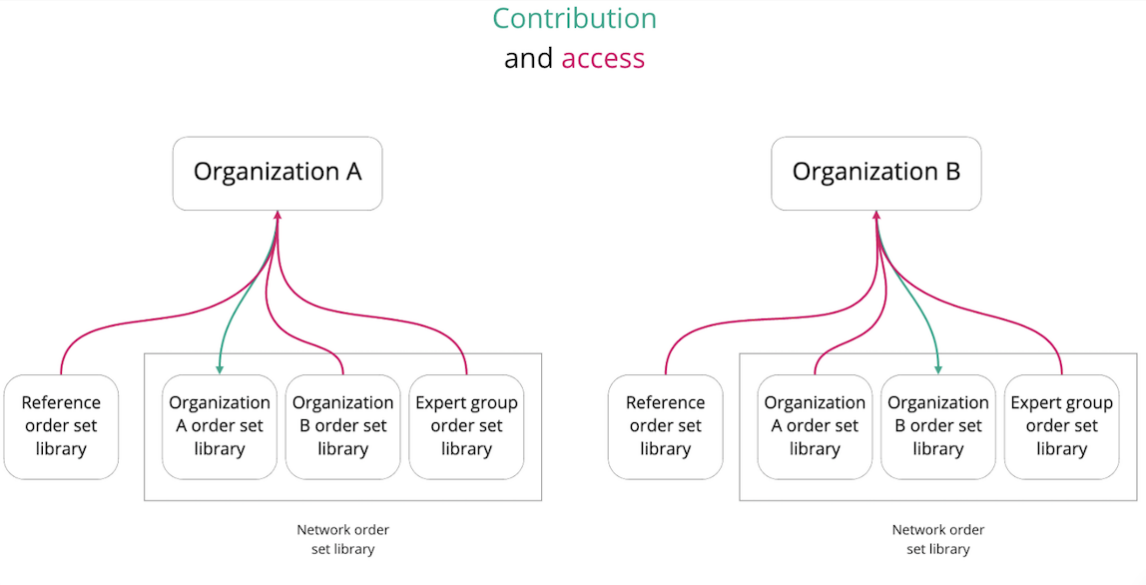
Contribution and access activity on the collaborative network, simplified using a model with only 2 organizations.

Users of TCN log in to the application and search for order sets through the reference order set library, network order set library, or their organization’s order set library (Figure 1). The reference order set library contains order sets that are developed by the Think Research Clinical Research and Development team, which consists of physicians, nurse practitioners, nurses, pharmacists, and other clinicians. The network order set library contains all of the order sets that are developed by partner agencies (eg, governmental and clinical specialist groups) and local participating hospitals or health care institutions. Depending on the type of order set the user is interested in, the user would select the relevant library, and results can be refined by filtering with diagnosis (eg, community-acquired pneumonia), hospital location (eg, emergency department), and other keywords. The user also has the option to use the search field (queries are made by title and keyword).

### Ethics Approval

As this study was considered a program evaluation, it did not fall under the auspices of the research ethics board and was exempt from research ethics board review. In addition, the study was considered as using data as a secondary use that does not involve any identifier information that is specific to an individual.

### Study Setting and Participants

Metadata for all participating Canadian hospitals and institutions, including hospital name, location, and number of beds, were available on the centralized network. These data were collected for all participating Canadian hospitals and institutions accessing the platform from January 2015 to March 2020. Although various countries make use of the platform, we sought to describe the network in a Canadian context. Data not pertaining to Canadian users were removed from our analysis. Hospital and organization names were anonymized to preserve hospital confidentiality.

### Data Collection

**Figure 2:**
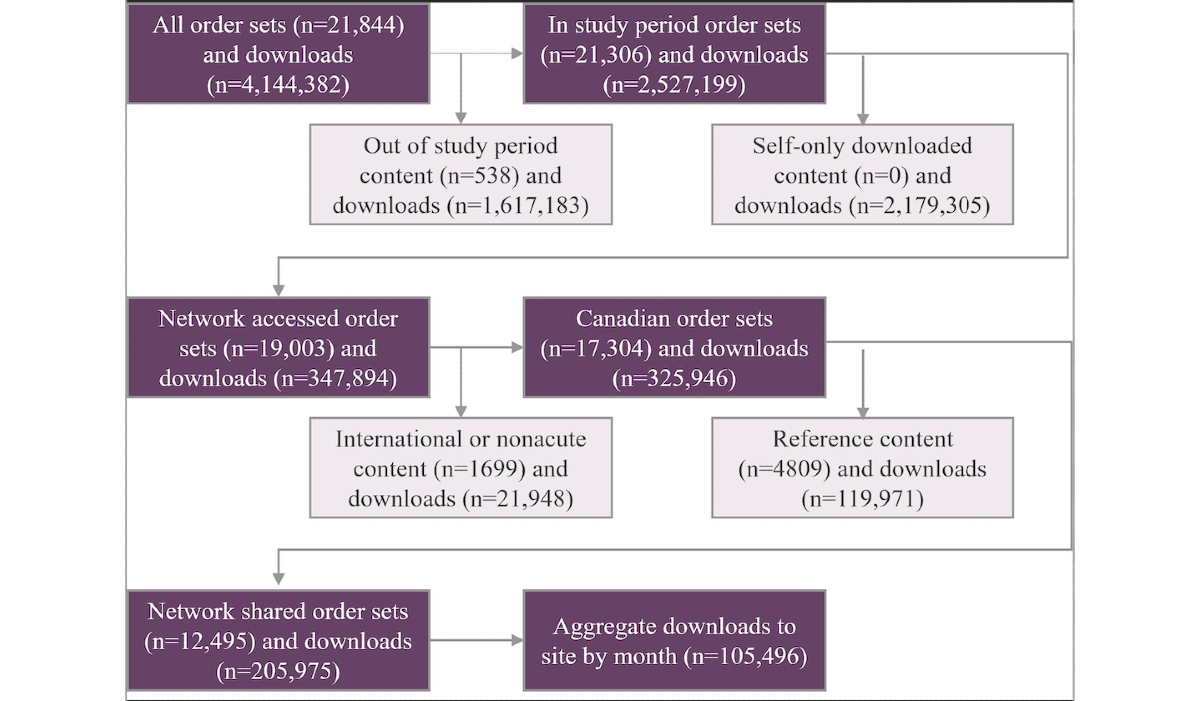
Data selection criteria and associated number of resources for the final data set.

Filters were sequentially applied to the entirety of the data set to identify eligible order sets. These selection criteria were applied broadly across the entirety of TCN or order set library. A summary of the selection criteria for order set selection is displayed in Figure 2. As our intention was to understand interorganizational behavior, we excluded intraorganizational downloads. We also did not include those downloads from the reference library (ie, Think Research) to highlight sharing among health care organizations instead of content downloads from the reference library.

We collected metadata for all downloaded content, including order set title and document, associated content downloads (eg, clinical protocols and supporting documents), downloading hospital or institution, and owner hospital or institution. All order sets and supporting documents were coded into either a medical diagnosis or medical process, if a diagnosis was not applicable. Hospitals were categorized as small (≤70 beds), medium (> 70 beds but <300), large (>300 beds), or as *group* if the organization consisted of more than one hospital or health care institution.

### Descriptive Analysis

This study focused on understanding the broad patterns of network behavior across organizations. To that end, we focused on understanding trends in the types of content shared at the level of diagnosis and institution so as to avoid inferences about specific institutional trends. To reduce the impact of multiple downloads from a site over a short period by the same user, we aggregated all downloads within a month by a single user at an institution into a single action per month.

We aimed to describe overall content availability, lifetime highly shared content, and active content (defined by the number of shares over the lifetime of the content). We also examined the properties of the hospital institution’s origin and downloader to describe high-level trends in sharing.

### Network Analysis

We also aimed to understand if variances in institutional size were related to a site’s tendency to be a source (provider of the order set) or sink (user of the order set) for content. We chose not to include each institution’s health information system or geography to preserve anonymity in the analysis. We performed a weighted chi-square analysis, where the expected number of downloads was estimated by the cross-product of content available at each hospital size by the total number of downloads of content. A post hoc analysis was conducted using pairwise comparisons corrected using Bonferroni adjustment. Estimated relationship strength was calculated using Cramer V.

Descriptive statistics were computed using Excel (Microsoft Excel version 16.52). Statistical analysis and network graphs with nodes were generated using R (R version 3.6.1).

## Results

### Descriptive Analysis

The participants of TCN consisted of 131 unique health care organizations (including hospital groups), consisting of 360 hospitals and institutions across 8 provinces in Canada. Hospital organizations ranged in size from 11 to 3119 beds with a median number of 134 beds per hospital and a mean of 319.9 (SD 514.8) beds (Table 1). The total data set represents approximately 45.89% (41,906/91,325) of the total beds staffed in Canada [24]. Seven institutions did not share or download content from the platform and were excluded from the study.

During the study period, 12,495 institutional order sets were generated and shared by the participating institutions (Figure 2). Collectively, these order sets correspond to 658 unique medical categories and diagnoses (eg, congestive heart failure and acute kidney injury) and 25 hospital processes (eg, intensive care unit admission). The 10 most commonly shared order set diagnoses represented 21% (22,150/105,496) of all shares (Table 2). Adjusting for the length of time content was available on the network, and rare content (ie, <5 order sets per diagnosis), content relevant to COVID-19, was among the most actively shared content (Table 3).

**Table 1:** Characteristics of participant health care organizations (N=131).

Health care organization characteristic		Health care organizations, n (%)
**Participant health care organizations**		
	Small (≤70 beds)	44 (33.6)
	Medium (>70 but ≤300 beds)	40 (30.5)
	Large (>300 beds)	25 (19.1)
	Hospital group (>1 hospital^a^)	22 (16.8)
**Geography**		
	Alberta	0 (0)
	British Columbia^b^	2 (1.5)
	Manitoba^c^	1 (0.8)
	New Brunswick	0 (0)
	Newfoundland and Labrador^d^	4 (3.1)
	Nova Scotia^e^	2 (1.5)
	Ontario^f^	110 (84.0)
	Prince Edward Island^g^	1 (0.8)
	Quebec^c^	1 (0.8)
	Saskatchewan^h^	10 (7.6)
	Northwest Territories	0 (0)
	Nunavut	0 (0)
	Yukon	0 (0)

^a^251 hospitals are part of a larger hospital group.

^b^37 hospitals.

^c^1 hospital.

^d^34 hospitals.

^e^38 hospitals.

^f^138 hospitals.

^g^7 hospitals.

^h^104 hospitals.

**Table 2:** Most available order sets and most downloaded order sets by diagnosis.

Order set category		Order sets, n (%)
**Content available**		12,495 (100)
	Acute coronary syndrome	359 (2.87)
	Stroke or TIA^a^	284 (2.27)
	Analgesia	273 (2.18)
	Venous thromboembolism	218 (1.74)
	Diabetes	205 (1.64)
	Labor	185 (1.48)
	COPD^b^	182 (1.46)
	Asthma	178 (1.42)
	CHF^c^	177 (1.42)
	Palliative care	159 (1.27)
	Other disease conditions	10,275 (82.23)
**Total downloads over lifetime**		105,496 (100)
	Acute coronary syndrome	4122 (3.91)
	Analgesia	3319 (3.15)
	Venous thromboembolism	2696 (2.56)
	Alcohol use, detoxification, and withdrawal	1794 (1.70)
	Palliative care	2386 (2.26)
	Diabetic ketoacidosis	1891 (1.79)
	Stroke or TIA	832 (0.79)
	Labor	1854 (1.76)
	Total parenteral nutrition	1740 (1.65)
	Diabetes	1516 (1.44)

^a^TIA: transient ischemic attack.

^b^COPD: chronic obstructive pulmonary disease.

^c^CHF: congestive heart failure.

**Table 3:** Most active content over lifetime (N=12,495 order sets).

Diagnosis group	Order sets, n (%)	Years available, mean (SD)	Absolute activity^a^
Acute coronary syndrome	359 (2.87)	2.68 (1.44)	2092.77
Stroke or TIA^b^	284 (2.27)	1.93 (1.46)	1866.64
COVID-19	22 (0.18)	0.08 (0.04)	1470.26
Analgesia	273 (2.18)	2.88 (1.43)	1400.96
COPD^c^	182 (1.46)	2.03 (1.36)	1291.47
Venous thromboembolism	218 (1.74)	2.81 (1.45)	1213.54
Alcohol use, detoxification, and withdrawal	156 (1.25)	2.70 (1.38)	1057.76
Diabetic ketoacidosis	205 (1.64)	2.58 (1.52)	1012.35
Diabetes	148 (1.18)	2.28 (1.48)	1005.66

^a^The sum of all downloads of content within that diagnosis divided by the total lifetime of all content within that diagnosis group.

^b^TIA: transient ischemic attack.

^c^COPD: chronic obstructive pulmonary disease.

### Network Analysis

**Figure 3:**
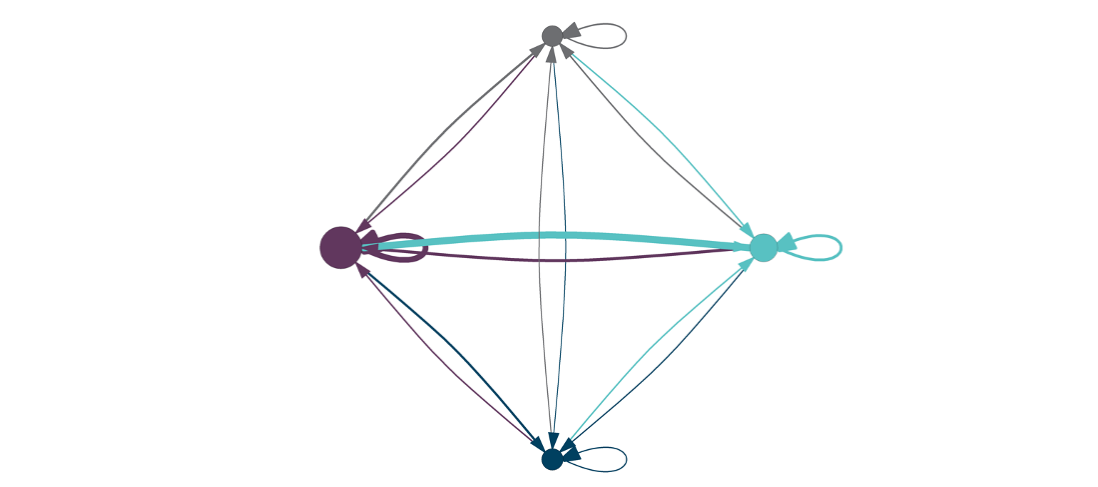
Network diagram showing total collaborative network for downloads of order sets during the study period by hospital size. Node color represents hospital size (gray=small; teal=medium; purple=large; loyal blue=group). Node size is representative of the source size of the institution (ie, popularity of that site’s content). Edge color and width represent sink hospital size and the relative number of downloads (ie, thicker=more unique downloads of content). Arrows point from source hospital to sink hospital.

**Figure 4:**
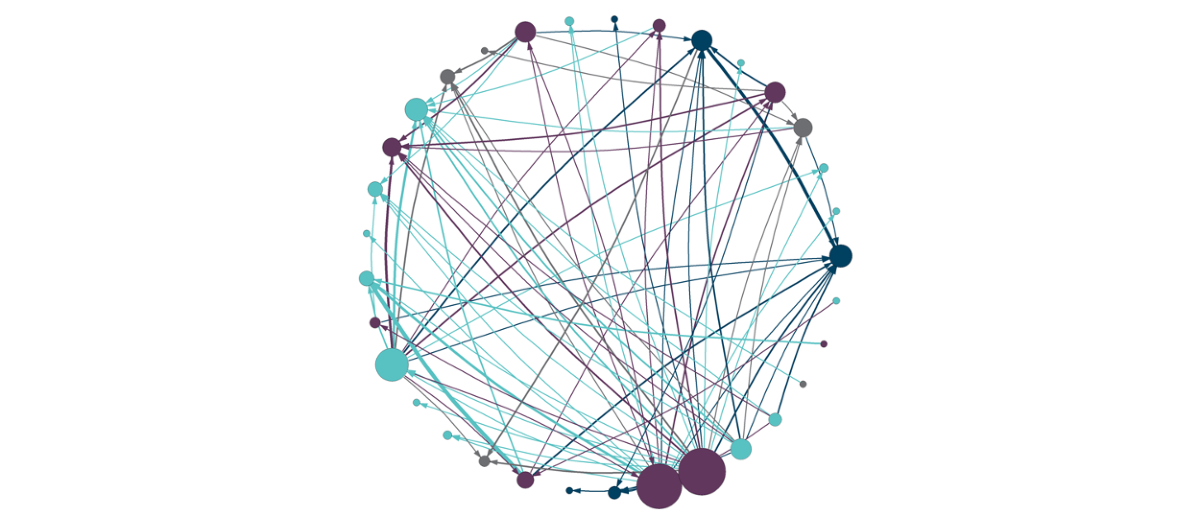
Network graph of order set downloads for order sets corresponding to COVID-19. Node color represents hospital size (gray=small; teal=medium; purple=large; loyal blue=group). Node size is representative of the source size of the institution (ie, popularity of that site’s content for this particular order set). Edge color and width represent sink hospital size and the relative number of downloads (ie, thicker=more unique downloads of content). Arrows point from source hospital to sink hospital.

There were a total of 105,496 shares among institutions between January 2015 and March 2020. Institutions shared on average 98.8 distinct order sets a mean of 574.0 times (median 200; range: 0-5371). Institutions downloaded a mean of 517.8 unique order sets (median 294; range 0-5342) for a mean of 718.9 times (median 367; range: 0-7316; detailed breakdown available in Multimedia Appendix 1). Content was available for an average of 2.32 years (median 2.32 years; range 0.08-5.25 years). Figure 3 demonstrates a network for the sharing of all order sets stratified by hospital size. Figure 4 demonstrates a network for sharing of order sets related to COVID-19 as a specific example to highlight the sharing among sites.

At the institution level, there was a significant (weighted *χ^2^*_15_=64313.9; *P*<.001) difference between the expected number of downloads by hospital size and content availability (Table 4). Medium-sized institutions downloading content from large institutions were the largest drivers of this variance (chi-gram=124.70). Post hoc analysis showed that all institution size interactions were statistically significant (Table 4), with a moderate interaction between institution size of order set providers and users (Cramer V=0.26).

**Table 4:** Summary of institutional sharing and content availability of 105,496 shares.

Downloader		Owner			
		Small	Medium	Large	Group
**Small **					
	Shares, n (%)	4391 (4.16)	4046 (3.84)	6075 (5.76)	2782 (2.64)
	Chi-gram	6.72^a^	–18.66^a^	–12.13^a^	–23.13^a^
**Medium**					
	Shares, n (%)	4386 (4.16)	8518 (8.07)	21029 (19.93)	4044 (3.83)
	Chi-gram	–9.98^a^	18.60^a^	124.70^a^	–19.92^a^
**Large**					
	Shares, n (%)	4462 (4.23)	9114 (8.64)	16906 (16.03)	4632 (4.39)
	Chi-gram	–32.07^a^	–7.01^a^	35.86^a^	–35.68^a^
**Group**					
	Shares, n (%)	2074 (1.97)	3613 (3.42)	6135 (5.82)	3289 (3.12)
	Chi-gram	–29.16^a^	–23.39^a^	–9.94^a^	–14.31^a^

^a^*P*<.00006 (Bonferroni adjusted *P*<.001).

## Discussion

### Principal Findings

We have presented a description and analysis of a Web 2.0 platform that facilitated the sharing of order sets across Canada, over a breadth of hospital sizes and clinical specialties, with >100,000 shares across 131 unique health care organizations over 5 years. This is the first study of its kind that has described the sharing of clinical decision supports, and more broadly, translation of executable knowledge at this scale with transparency. Although other studies have focused on artifacts of exchange (ie, amount of content available), this study also demonstrates the actual network of sharing supported by content [9].

Various models have been proposed to conceptualize knowledge translation. One framework developed by Graham et al [25,26] and subsequently adopted by the Canadian Institutes of Health Research defines knowledge translation as a dynamic and iterative process concerning the creation, dissemination, and application of knowledge. The foundation of this framework proposed by Graham et al [25,26] involves knowledge creation, consisting of knowledge inquiry (eg, primary research concerning a particular disease state), synthesis (eg, systematic reviews and guidelines derived from primary research), and then products or tools (eg, order sets developed by an entity such as Think Research, which is then disseminated). Knowledge transfer, the primary focus of TCN, is a fundamental step in the knowledge translation process. Central to knowledge translation is also the adoption of knowledge in the local context from a top-down perspective. This can occur with order sets as well.

### Order Set Customization and Sharing

Order set customization may be done for multiple reasons, including local variations in care processes, differences in resource availability (eg, a 14-bed hospital will have different resources than a 1500-bed hospital), or ambiguity in high-level recommendations provided in clinical guidelines (eg, keeping systolic blood pressure below 160 mm Hg under certain circumstances but not specifying which drugs to use and when to start). In addition, many care processes are not always addressed in the guidelines and other medical literature. For this type of content, organizations must create their own order sets. These mechanisms all contribute to the customization of order sets and produce the variations that exist among organizations. In turn, this variation can catalyze sharing.

The most significant finding of this study is the heavy use of the network for the purpose of knowledge transfer. There were no mechanisms in place to enforce or encourage sharing of order sets. Users of the platform could opt to, for example, download a complete order set from the reference library instead of downloading from another institution. Despite this, there was extensive sharing. This may derive from a number of factors; for example, clinicians may wish to emulate the practice of a colleague, learn from the order sets of large academic centers, or inquire into how an order set was modified in a hospital with similar capacities and facilities.

Viewing another organization’s order sets takes time and effort from otherwise busy health care practitioners. Health care providers would not do this if they did not derive value from viewing another organization’s order sets. This downloading even occurred during a time of severe organizational stress during the early phases of the COVID-19 pandemic. Network activity also reflected these contemporary developments. For example, COVID-19 was first identified in Canada as early as January 23, 2020 [27]. However, as of March 2020, order sets related to COVID-19 have held a spot among the most actively shared order sets. This is telling of the network’s capacity for rapid knowledge transfer and, more broadly, knowledge translation.

An examination of sharing patterns stratified by hospital size also revealed that although the largest content source was large hospitals, contributing to 47.4% of shares, organizations of all sizes played substantial roles in sharing. This reflects the value that all organizations, regardless of size, can contribute to a network of knowledge and sharing.

Although users of TCN had no explicit measures or incentives to do so, the sharing of order sets was the predominant mechanism of knowledge translation. This may suggest that the mere availability of the platform naturally promoted sharing. This trend of increasing uptake of Web 2.0 platforms as a means of knowledge translation among clinicians has also been seen with platforms such as Twitter and blogs [15]. As technologies continue to evolve and clinicians become increasingly interconnected, strategies for knowledge translation must adapt appropriately.

Although this study sought to document the overall trend in order set sharing across a network, it does not attempt to describe the reasons for content being accessed. There are a number of possibilities, including organizations reusing the entire order set for their own local practice, reusing components and reintegrating it into another order set, or simply using it as a general source of knowledge or ideas. In addition, although we do examine how resources were disseminated and exchanged among participants in the network, we do not examine exactly how these order sets were used in the clinical setting (eg, the proportion of downloads that led to an order set being used in the clinical setting and whether they were understood or used properly). Although knowledge transfer is a critical part of knowledge translation, implementation of knowledge in a clinical setting is also important to evaluate. Finally, there were other factors for analysis that could be evaluated, including geography, electronic health record (EHR) system, and user-specific access. Further study in these areas could produce valuable insights about the outputs as a result of the sharing of executable knowledge.

### Next Steps

Looking forward, physician learning and knowledge translation will likely continue to harness the potential of Web 2.0. The response of the academic and clinical landscape to COVID-19 represents a prime example of how knowledge translation systems must adapt to the need for rapidly changing information. Platforms such as Slack and Twitter served as mechanisms to evaluate and distribute preprints of articles and processes that accelerated knowledge translation [28]. Twitter, WeChat, and university websites also served as platforms for sharing infographics and expert recommendations [29,30]. Much of the information disseminated through these means were referential in nature (eg, disease characteristics and epidemiology), with less translation of experiential knowledge or tools that could be applied for on-the-job learning [31].

Similarly, pre-existing platforms, such as information systems and EHRs, can continue to evolve to support better care plans, improve interdisciplinary communication and workflow, and provide clinical decision supports. In this process, order sets may play an increasingly important role in EHRs. The *plan-centric* EHR is forward looking and focuses on redesign to not only record patient information but also to enhance patient care and optimize the delivery of care. Seamless adoption of order sets into EHRs may be one component of this redesign; the ability to access localized order sets across geographic barriers can serve as an accelerant for the consistent integration of evidence-based guidelines in future EHR design [32].

As Web 2.0 platforms continue to evolve, ethical use must also be considered. The benefits of mass user contributions, convenience, and low barriers to entry of using Web 2.0 platforms must be considered in light of some of the possible drawbacks, such as lack of peer review and the possibility of the spread of misinformation [30,33,34]. To this end, guidelines have been developed by authors for the responsible use of social media–disseminated information [30]. In addition, these growing platforms should be viewed not necessarily as a replacement of more traditional knowledge translation activities but as a supplement. Synergistic benefits can manifest from marrying various types of platforms, producing gains for physician collaboration, CME, and enhancing experiential learning through sharing of executable knowledge.

## Appendix

Multimedia Appendix 1 Order set availability and access by institution.

## Abbreviations

CME: continuing medical education
EHR: electronic health record
TCN: the collaborative network
