# Supplementary material for: Examination of a Canada-Wide Collaboration Platform for Order Sets: Retrospective Analysis
Source: J Med Internet Res. 2021 Nov 29;23(11):e26123. doi: 10.2196/26123 (PMC8669583; doi:10.2196/26123)
Supplement: Multimedia Appendix 1 [file jmir_v23i11e26123_app1.docx]

Table i. Order set availability and access by institution.

| Institution Size | Mean (SD); Median (Min - Max) |
| --- | --- |
| Owner - Shared content  Small  Medium  Large  Group  Total | 413.86 (597.85); 167 (7 - 2895)  766.39 (767.73); 527 (3 - 3021)  2387.86 (2510.92); 1900 (24 - 9402)  867.47 (864.5); 505 (1 - 3088)  976.81 (1450.05); 452 (3 - 9402) |
| Owner - Unique order sets  Small  Medium  Large  Group  Total | 46.65 (52.82); 25 (4 - 274)  97.61 (93.11); 72 (2 - 393)  262.95 (248.76); 213 (6 - 895)  119.18 (122.55); 87 (1 - 440)  115.69 (152.81); 54.5 (1 - 895) |
| Owner - Shared diagnoses  Small  Medium  Large  Group  Total | 29.81 (27.27); 18 (2 - 134)  55.52 (42.95); 46 (2 - 160)  109.19 (80.59); 115 (6 - 247)  58.12 (49.66); 44 (1 - 173)  57.56 (56.35); 40.5 (1 - 247) |
| Owner - Average life of order set on network  Small  Medium  Large  Group  Total | 2.15 (0.86); 2.29 (0.76 - 4.19)  2.26 (0.78); 2.26 (0.29 - 3.83)  2.28 (0.75); 2.54 (0.68 - 3.15)  2.08 (0.67); 2.26 (0.51 - 2.94)  2.20 (0.78); 2.28 (0.29 - 4.19) |
| User - Downloads  Small  Medium  Large  Group  Total | 443.44 (1068.74); 182 (7 - 6618)  999.39 (1356.93); 479 (7 - 7316)  1463.08 (1850.05); 765.5 (1 - 6750)  795.21 (798.31); 610 (2 - 2349)  879.12 (1353.47); 382 (1 - 7316) |
| User - Unique order sets downloaded  Small  Medium  Large  Group  Total | 328.31 (842.66); 151 (7 - 5342)  654.68 (627.16); 425 (7 - 2274)  1000.62 (1126.99); 614.5 (1 - 3726)  597.37 (568.06); 493 (2 - 1627)  608.73 (837.13); 297 (1 - 5342) |
| User - Unique diagnoses  Small  Medium  Large  Group  Total | 59.62 (74.51); 39 (3 - 442)  123.18 (93.64); 96.5 (6 - 376)  152.33 (124.46); 144 (1 - 379)  113.21 (90.41); 100 (1 - 247)  106.78 (99.85); 76 (1 - 442) |
